# Climate change may alter seed and seedling traits and shift germination and mortality patterns in alpine environments

**DOI:** 10.1093/aob/mcaf132

**Published:** 2025-06-23

**Authors:** Jerónimo Vázquez-Ramírez, Susanna E Venn

**Affiliations:** School of Life and Environmental Sciences, Deakin University, 221 Burwood Hwy, Melbourne, Victoria 3125, Australia; School of Life and Environmental Sciences, Deakin University, 221 Burwood Hwy, Melbourne, Victoria 3125, Australia

**Keywords:** Decrease in precipitation, fire, manipulative experiment, seeds, seedlings, warming

## Abstract

**Background and Aims:**

The early life-history stages of alpine plants are strongly influenced by climatic factors. Predicted changes in alpine climate could significantly impact the ability of plants to regenerate from seed and thus on the long-term survival of these species. However, our knowledge on this topic has focused predominantly on the effects of warming on germination. Other early life-history stages or other changing environmental factors have been overlooked. Here, we determine the effects of a future warmer and drier climate and post-fire conditions on (1) seed development, (2) germination and (3) seedling establishment of 13 alpine species.

**Methods:**

For 2 years, we conducted a factorial field-based manipulative experiment in the Australian Alps where we created a warmer and drier microclimate using modified open-top chambers and post-fire conditions by controlled burning of selected plots. We (1) collected seeds that had developed under experimental conditions and determined their mass, size, germinability and the characteristics of emerging cotyledons; (2) buried seeds in experimental plots and monitored germination throughout the snow-free season; and (3) grew and planted seedlings and determined their survival over a year.

**Key Results:**

Overall, we found negative responses to the experimental treatments. Warmer and drier conditions during (1) seed development reduced seed mass, seed size and cotyledon area. (2) Seed germination decreased in response to warmer and drier and post-fire conditions. Importantly, the timing of germination shifted under warmer and drier conditions. (3) Seedling establishment was negatively affected by post-fire conditions and, to a lesser extent, by warmer and drier conditions. The timing of seedling mortality shifted relative to the control conditions.

**Conclusions:**

Predicted future climate will have a negative impact on the ability of plants to regenerate from seed. Furthermore, species-specific responses are likely to contribute to changes in the composition and diversity of alpine communities.

## INTRODUCTION

While climate change poses a threat to all ecosystems around the globe, alpine ecosystems are among the most at risk. Their limited distribution (i.e. narrow elevation bands on mountains above the treeline and below the nival zone, <3 % of the global surface; Testolin *et al*., 2020, Körner, 2021) and a greater temperature increase in these regions compared to other parts of the globe (exceeding the global warming rate by 0.2 ± 0.1 °C per decade; Hock *et al*., 2019) make them particularly sensitive to global warming. Increasing temperatures are driving changes in other environmental factors, such as changes in precipitation patterns (Gobiet *et al*., 2014, Hock *et al*., 2019) and reductions in the depth and duration of snow cover (Sánchez-Bayo and Green, 2013, Hock *et al*., 2019). More recently, extreme climate-related events such as fires, historically rare in alpine environments, are becoming more frequent and intense in some regions, including parts of South America and Australia (Williams *et al*., 2008, Collins *et al*., 2022).

To date, there is considerable evidence that climate change has affected several aspects of alpine plant life, such as distribution (Parolo and Rossi, 2008, Pauli *et al*., 2012, Lamprecht *et al*., 2018, Auld *et al*., 2022), phenology (Molau *et al*., 2005, Smith *et al*., 2012, Inouye, 2019) and reproduction (Mondoni *et al*., 2012, Hoyle *et al*., 2014, Rosbakh *et al*., 2017). Of these, reproduction via seed is likely to exhibit more rapid and pronounced responses to climate change, as early life-history stages, such as seed development, germination and seedling establishment, are directly influenced by changing environmental conditions (Walck *et al*., 2011, Körner, 2021). Despite their importance, our understanding of how these early stages respond to ongoing environmental changes remains poorly understood (Parmesan and Hanley, 2015).

During the seed development stage, reproductive output is shaped by the availability of resources (e.g. water, nutrients) and environmental conditions (e.g. temperature; Körner, 2021). Previous research has shown that higher temperatures and a longer growing season can increase seed production and mass in alpine species (Wagner and Reichegger, 1997, Wirth *et al*., 2010). Water limitation, in contrast, tends to reduce or have neutral effects on seed quantity and size (Rosbakh *et al*., 2017). While existing studies have examined the effects of warming or water availability on seed mass individually, these factors are increasingly co-occurring under climate change and may interact in complex ways to influence reproductive outcomes.

The ‘germination niche’ of alpine species is characterized by physiological dormancy, a strong need for cold stratification and positive responses to high soil moisture and higher temperatures (Fernández-Pascual *et al*., 2021). Therefore, expected changes in alpine climates will certainly affect germination. Higher temperatures can increase germination rates and accelerate germination (Milbau *et al*., 2009, 2017, Mondoni *et al*., 2012, Hoyle *et al*., 2013, Fernández-Pascual *et al*., 2021), while reductions in soil moisture can suppress germination (Walder and Erschbamer, 2015, Rosbakh *et al*., 2022, Gya *et al*., 2023) and alter dormancy-breaking processes (Chen *et al*., 2022). Extreme climate-driven events such as fire may also influence germination, promoting it in some fire-tolerant species while reducing it in other fire-sensitive ones (Vázquez-Ramírez and Venn, 2023).

Seedling establishment is a critical recruitment bottleneck and the stage most vulnerable to failure (Fenner and Thompson, 2005). Newly emerged seedlings are exposed to the harsh alpine environment and must quickly grow roots into the soil or die from topsoil desiccation, heat and soil freeze/thaw cycles (Körner, 2021). Warming has shown mixed effects on establishment, with some studies reporting increased survival (Mondoni *et al*., 2015, Milbau *et al*., 2017, Wang *et al*., 2018) and others documenting declines (Graae *et al*., 2009, Shevtsova *et al*., 2009, Meineri *et al*., 2013). Although increased moisture is generally thought to improve establishment (Forbis, 2003, Shevtsova *et al*., 2009), some species show a weak or no response to additional water (Meineri *et al*., 2013). In contrast, reduced water availability consistently has negative effects on seedlings (Venn and Morgan, 2009, Gya *et al*., 2023). Additional environmental changes, such as fires, can expose seedlings to altered microclimatic conditions that may shift community composition and reduce species diversity (Camac *et al*., 2017).

Despite our growing understanding of the responses of alpine seeds and seedlings to changing environmental factors, important knowledge gaps remain. Reproductive stages and environmental variables have been studied unequally, with most research focused on higher temperatures and germination (Vázquez-Ramírez and Venn, 2021, Mondoni *et al*., 2022). For other stages, such as seed development and seedling establishment, and for drivers like changes in precipitation and extreme events such as fire and droughts, our understanding remains very limited and often comes from a single study or region (Vázquez-Ramírez and Venn, 2021, Mondoni *et al*., 2022). Furthermore, only 40 % of existing studies have assessed the combined effects of warming with other environmental changes despite their expected co-occurrence (Vázquez-Ramírez and Venn, 2021). Considering that warming may simultaneously alter water availability, snowpack and fire regimes, experiments that combine multiple stressors are essential for predicting alpine plant responses to climate change.

Here, we address some of these gaps using a 2-year field manipulative experiment to investigate the effects of a future warmer and drier alpine climate, in combination with post-fire conditions (two stressors expected to become more frequent and co-occur in the future; Williams *et al*., 2008, Collins *et al*., 2022), on (1) seed development, (2) seed germination and (3) seedling establishment of 13 Australian alpine species. The specific research questions are: (1) What are the effects of a warmer and drier environment during seed development on alpine seed and seedling traits? (2) How will a future warmer and drier environment, in combination with post-fire conditions, affect germination time and germination rates in alpine environments? (3) How will the seedling establishment of alpine species be affected by future increasing temperatures, reduced water availability and post-fire conditions?

## METHODS

### Study area and species

**Fig. 1.**
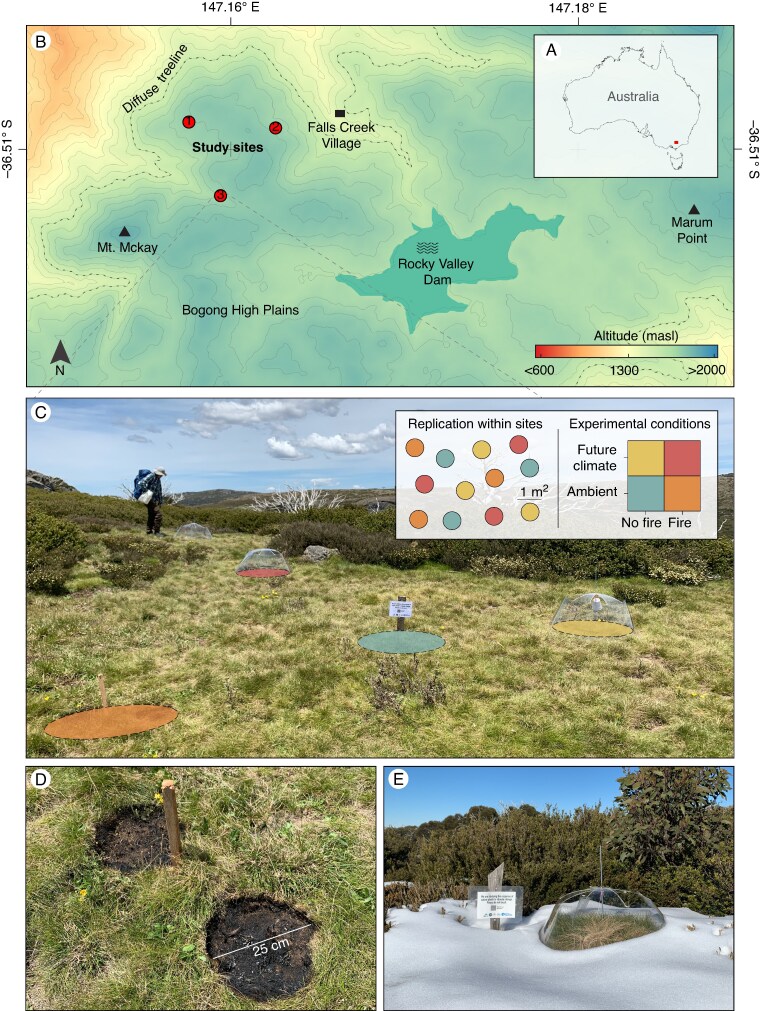
Locations of Bogong High Plains within Australia (A) and study sites within the Bogong High Plains (B), experimental design within each site and experimental site showing some of the established plot (C), detail of fire treatment (D) and OTC after snow shower (E).

The study took place in the Bogong High Plains, Victoria, south-eastern Australia (Fig. 1A) during two Austral snow-free periods: November 2020–May 2021 and November 2021–May 2022. Mean annual temperature is 5.3 °C, with a mean maximum temperature for the hottest month (January) of 17.8 °C and a mean minimum temperature for the coldest month (July) of −2.9 °C (1990–2023). Mean annual precipitation is 1359 mm (1990–2023), with most of the precipitation falling as snow in winter, which can persist for around 4 months, and the driest period of the year being summer (Falls Creek meteorological station, Bureau of Meteorology, www.bom.gov.au). The soils are free-draining highly acidic alpine humus (Costin *et al*., 2000).

Thirteen species were selected for the study (Table 1). They are common components of the tall alpine herbfield (*sensu* Costin *et al*., 2000). The chosen species have different regeneration strategies after fires and are of different plant life forms. Although we intended to study all species during the three life stages of interest (i.e. seed development, seed germination and seedling establishment), unexpected circumstances, such as poor seed production within the experimental plots, made it impossible to study the seed development stage in six species (Table 1). Space limitations within the manipulative experiment also meant that each species was only studied in one of the two experimental periods.

**Table 1.** Study species, family, life form (LF; G = graminoid, F = forb, S = shrub), main regeneration strategy after fire (RS; S = seeder, R = resprouter) and life stage under which they were successfully studied (•).

Species	Family	LF	RS	Life stage		
				Seed development	Seed germination	Seedling establishment
*Carex breviculmis*	Cyperaceae	G	R	•	•	•
*Luzula modesta*	Juncaceae	G	R	•	•	
*Poa hothamensis*	Poaceae	G	R	•	•	•
*Rytosperma nudiflorum*	Poaceae	G	R	•	•	•
*Oreomyrrhis eriopoda*	Apiaceae	F	S		•	•
*Brachyscome spathulata*	Asteraceae	F	S		•	•
*Celmisia pugioniformis*	Asteraceae	F	R		•	•
*Craspedia aurantia*	Asteraceae	F	R	•	•	•
*Erigeron bellidioides*	Asteraceae	F	S	•	•	•
*Acetosella vulgaris* *	Polygonaceae	F	R	•	•	•
*Ranunculus victoriensis*	Ranunculaceae	F	S	•	•	•
*Stylidium armeria*	Stylidiaceae	F	S		•	•
*Olearia frostii*	Asteraceae	S	S	•	•	•

^*^Exotic species.

### Experimental design

Within the study area, we selected three sites with similar environmental and topographic conditions, including aspect, slope, soil properties and species composition (Fig. 1B). At each site, we established 12 circular plots of 1 m^2^ with predominantly short-statured vegetation (<20 cm tall) and where our target study species were present. Then, using a 2 × 2 factorial design with two levels of climate (control and future climate) and two levels of fire treatment (unburned and burned), plots were randomly assigned in equal numbers to one of four treatments: (1) control, (2) future climate, (3) fire and (4) future climate combined with fire (Fig. 1C). In total, our experiment included 36 plots (12 per site × 3 sites), with nine plots assigned to each experimental condition (3 per site × 3 sites).

**Fig. 2.**
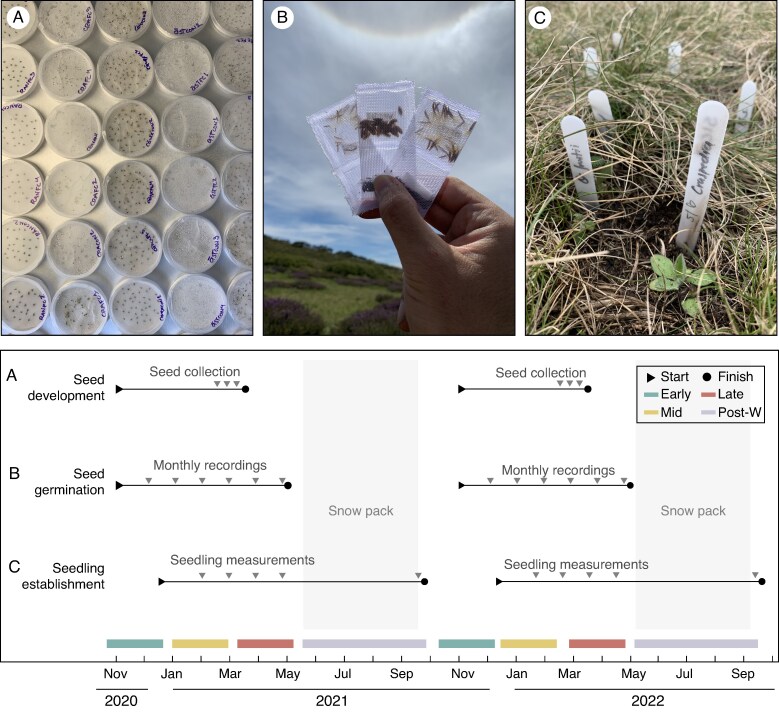
Experiment timeline and illustration of some of the methods used for the study of (A) seed development, (B) seed germination and (C) seedling establishment.

The future climate treatment simulated the higher temperatures and reduced soil moisture projected for the study region under climate change (Sánchez-Bayo and Green, 2013). The treatment was imposed using modified open-top chambers (OTCs) based on the traditional cone-shaped OTC (Marion *et al*., 1997), with the addition of rain-out structures to form a partially water-shedding roof (Fig. 1C, E). The chambers were made of 0.8-mm-thick clear flexible polycarbonate (Suntuf, Palram Industries, Ramat Yohanan, Israel) and had a base diameter of 84.6 cm, an open-top diameter of 50 cm, a height of 40 cm and a 50 % rain restriction. Additional information on the modified OTCs can be found in Vázquez-Ramírez and Venn (2024). To hydrologically isolate and prevent subsurface water flow into and out of the chambered plots, we buried garden edging (rigid but flexible plastic, 1 mm thick and 10 cm high) up to 10 cm below the soil surface around the selected plots but within the chamber area. The chambers were installed 1 week before the start of each experimental year (see Fig. 2) and removed during the winter to avoid damage from snow accumulation. In the second year, the chambers were reinstalled in the same plots within each site.

The fire treatment was designed to simulate the heat and smoke chemicals generated during a wildfire. It consisted of a prescribed mini-burn on the selected plots using a hand-held propane safety torch contained within the sides of a 25-cm-diameter metal ring. Two rings were burned in each fire-plot: one to test germination and another to test seedling establishment (Fig. 1D). The treatment simulated a low-intensity fire that burned all loose litter and vegetation within the selected area. After the burning, additional smoke chemicals were applied by watering the plots with 500 mL of a 5 % concentration solution of smoke water (Regen 2000, Grayson, Australia). The no-burn plots were also watered, but with 500 mL of plain water to keep water inputs consistent. The fire treatment was applied in mid-spring 2020 and 2021, on the same day that seeds were buried in the experimental plots (see Fig. 2).

### Micro-climate monitoring

To determine the effects of our treatments on temperature and soil moisture throughout the study, we recorded hourly abiotic conditions in three randomly selected plots per site: one control, one future climate and one fire (*n* = 3 per condition). To infer soil moisture, volumetric water content (VWC) was recorded using HOBO H21-USB Micro Stations and HOBO-S-SMD-M005 sensors. Temperature loggers (HOBO MX2201) were installed at the soil surface and 1–3 cm below the soil surface. Light intensity was measured at noon (1200 h) at ground level from 1 to 31 January 2021 using a HOBO Pendant UA-002-64 data logger. Wind velocity was measured once with two Kestrel-1000 anemometers at 10 cm above the soil surface inside a chamber and in a control plot at 0900, 1200 and 1700 h (February 2022). Daily rainfall for the study period was obtained from the nearby Bureau of Meteorology weather station (1.5 km away).

### Seed development

To determine the effects of our treatments during the seed development stage, we collected seeds of our study species within our experimental plots (only future climate and control plots for this life-stage, since fire treatment burned the vegetation). Seeds were collected in late summer and early autumn (February–April of 2021 and 2022, Fig. 2) when they had darkened and were ready to disperse. After collection, seeds were stored in paper bags and transported to the laboratory. In the laboratory, seeds were cleaned, and dispersal structures were removed. Seed mass for each species and experimental treatment combination was determined by weighing five lots of 100 randomly selected seeds. Seed size was determined by analysing digital images; we photographed the five 100 seed-lots and then randomly measured the length of five seeds of each one using ImageJ software.

The germinability of the collected seeds was determined by a germination test. Prior to testing, seeds were wet-cold stratified for 8 weeks at 2 °C in dark conditions. For each species and treatment combination, five replicates of 30–50 seeds were placed on moist germination filter paper (Advantec Grade No. 101) in 90-mm Petri dishes and wrapped in parafilm. The dishes were then placed on a germination plate at a constant temperature of 25 °C with a 14/10-h light/dark cycle, with a light intensity of 2400 lux of visible light. Germination was recorded daily until no germination occurred after five consecutive days. Radicle emergence (>1 mm) was the criterion for a germinated seed. After the germination tests, any non-germinated seeds were tested with a cut test; seeds with a hard and white endosperm were considered potentially viable. Empty seeds were removed from the original count and from the analysis. New germinants were kept in the dishes until the cotyledons were fully open. We then measured cotyledon greenness using the Greenindex+ photographic application, which involved removing the new germinants from the Petri dishes and calibrating their colour against a template of known colour saturation. This application determines the DGCI (Dark Green Colour Index), a value between 0 and 1, that has been shown to be correlated to chlorophyll content (Gleadow *et al*., 2016). Finally, we measured cotyledon area using the same photographs and the ImageJ software.

### Germination

To determine the effects of the experimental treatments on seed germination, we collected seeds of the study species from alpine populations in Kosciuszko National Park in 2020, the Bogong High Plains (2020 and 2021) and Mt Hotham (2020). Seeds were collected from at least 30 individuals of the same population. In the laboratory, seeds were cleaned and temporarily stored in paper bags. The seeds were then wrapped in moist paper towels, sealed in air-tight plastic bags and cold stratified at 2 °C in dark conditions for 8 weeks.

After cold stratification, seeds were gently pressed against a hard surface to identify and exclude empty ones. The remaining seeds were randomly distributed in equal numbers across 36 samples (one per experimental plot). The samples, containing 25–50 seeds, depending on the species, were placed in plastic mesh bags (5 × 10 cm with a mesh size of 0.5 mm^2^, see Fig. 2). In mid-spring 2020 and 2021, after the snowpack melted in the experimental sites, we buried one randomly selected mesh bag per species to a depth of 1 cm in each experimental plot. During the subsequent snow-free seasons (November 2020–May 2021 and November 2021–May 2022, Fig. 2), we revisited the study plots and recorded monthly germination by removing the mesh bags from the soil, gently cleaning them with a brush, and holding them up to the light to count the number of seeds that had germinated (>1-mm radicle). The mesh bags were then returned to the soil. Each year, before the winter snow, we recovered all the mesh bags, opened them and counted the ungerminated seeds. We then matched these with germination records.

### Seedling establishment

A portion of the cold-stratified seeds collected prior to the experimental setup (see above) was used to grow seedlings for each of the study species. Seeds were sown into germination plugs (Jiffy, 36 mm) and watered under glasshouse conditions until seedlings developed at least two pairs of true leaves (dicot) or four leaves (monocot). Seedlings were then transferred to the experimental sites and acclimated to alpine conditions in the field for 1 week. Then, one seedling of each species was planted in each experimental plot in early December of each year, following seedling emergence patterns previously reported for the study area (Venn and Morgan, 2009). In the control and future climate plots, seedlings were randomly planted in natural free-vegetation gaps, without removing further vegetation. In the fire treatment, seedlings were planted directly into the experimentally burned patches (Fig. 2). Supplementary Data Database S1 shows the characteristics of the seedlings before being planted. Seedling survival (live/dead), seedling height (cm) and the number of leaves were recorded for each seedling at monthly intervals during the snow-free period and after snowpack melting (Fig. 2).

### Data analysis

During the data analysis, we looked for (1) general patterns (all species), (2) species-specific responses and (3) life form and regeneration strategy responses to our experimental treatments. For the last of these, we grouped species into different categories according to their life form (forbs, graminoids and shrubs) and their post-fire regeneration strategy (seeders, seed-based mechanism; and resprouters, vegetative mechanism) following McCarthy *et al.* (2003). Furthermore, the analysis was performed for three specific periods following the classification used by Venn and Morgan (2009). Periods correspond to natural soil temperature and moisture variations during the snow-free season in our study sites: (1) early period, wet and ‘warm’ temperatures – spring alpine conditions; (2) mid period, dry and ‘hot’ temperatures – typical summer alpine conditions; and (3) late period, wet and ‘cool’ temperatures – autumn conditions (Fig. 2). For the seedling establishment analysis, we added an extra period (winter period) corresponding to the time when we recovered the established seedlings after the snow cover melted (see ‘Germination’ section above, Fig. 2).

We used linear mixed models (LMMs) and generalized linear mixed models (GLMMs) to examine the effects of our treatments on the measured response variables. Models were fitted in R (R Core Team, 2022), using the package ‘lme4’ (Bates *et al*., 2015), and diagnostics and goodness-of-fit tests were performed using the package ‘DHARMa’ (Hartig, 2024). Data were log-transformed if they did not meet the assumption of normality (for LMMs). Figures were constructed using the ‘ggplot’ package (Wickham, 2016) and Microsoft Excel with further visual-only editing in Adobe Illustrator.

For the seed development stage, several LMMs with Gaussian-link were used to contrast seed mass, seed length, mean germination time, cotyledon area and cotyledon greenness between seeds produced in the different experimental treatments. In addition, GLMMs with a binomial distribution and logit-link were used to analyse germination. In the models, treatment (i.e. control or future climate) and life form or regeneration strategy were treated as a fixed effect and species as a random effect. Due to limited seed production during the study, seeds collected from plants in the same treatment but from different plots or sites were pooled, so we could not include the plot/site factor in the analysis. We then used the ‘emmeans’ package (Lenth, 2023) to perform *post hoc* tests, with confidence levels and *P*-value adjustments by Tukey corrections and to calculate the estimated marginal means. Species that failed to germinate (<5 % germination) were excluded from the germination and cotyledon analyses.

We analysed the final percentage of germination in the field using GLMMs with a binomial distribution and a logit-link for the complete study period and the above defined periods (i.e. early, mid, late). In the model, treatments (control, future climate, fire, future climate + fire) and their interaction with life form and regeneration strategy were treated as fixed effects, and species and plots nested within sites were included as random effects. To determine species-specific responses, we fitted a similar model but with species as a fixed effect and later performed *post hoc* tests, estimated marginal means and pairwise comparisons.

We contrasted seedling mortality and calculated survival probability with a GLMM with binomial distribution and logit-link. Treatments, life form and regeneration strategy were treated as fixed effects, and species and plots nested within sites as random effects. In addition, we fitted similar models to contrast the number of leaves and the height of the established seedlings before the winter of each year with a GLMM with negative binomial distribution and an LMM with Gaussian distribution, respectively. Species-specific responses were analysed by fixing the species in the model and performing *post hoc* tests. Finally, to explore factors contributing to high seedling mortality in the mid period, we fitted a GLMM with binomial distribution and logit link with treatments, leaf number, plant height, life form, regeneration strategy and year as fixed factors. Species and plots nested within sites were included as random effects.

## RESULTS

Overall, we observed negative and no effects of the experimental treatments on the measured response variables. Future climate treatment had negative effects on all three study life stages. Fire treatment had a weak negative effect on seed germination but a strong negative effect on seedling establishment.

### Experimental manipulations and microclimatic conditions

Ambient temperature and precipitation were similar in both years of the experiment (Supplementary Data Database S1). Future climate treatment (i.e. modified OTC) reduced the soil moisture by an average of 12.2 % during the experimental period and increased the number of days with soil moisture below the wilting point (Fig. S1). The chambers increased soil surface and subsurface temperatures by an average of 1.9 and 1.7 °C, respectively. Furthermore, the chambers significantly reduced the wind speed near the ground (+10 cm) at 0900 [*t*(4) = 9.67, *P* < 0.001], 1400 [*t*(4) = 10.93, *P* = 0.002] and 1900 h [*t*(4) = 13.05, *P* < 0.001]. There was a slight reduction in the relative light levels (lux) within the chambered plots compared to the controls, but the difference was not statistically significant [43 009 ± 2796 vs. 43 726 ± 3380, mean ± s.e.; *t*(55) = 0.49, *P* = 0.63].

Post-fire conditions (i.e. bare soil patches) did not affect soil moisture but increased soil surface and subsurface temperatures by an average of 0.51 and 0.5 °C, respectively, compared to control plots (Supplementary Data Table S1). Additional details on treatment effects and their variation throughout the snow-free season are provided in Table S1 and Fig. S1.

### Seed development

**Fig. 3.**
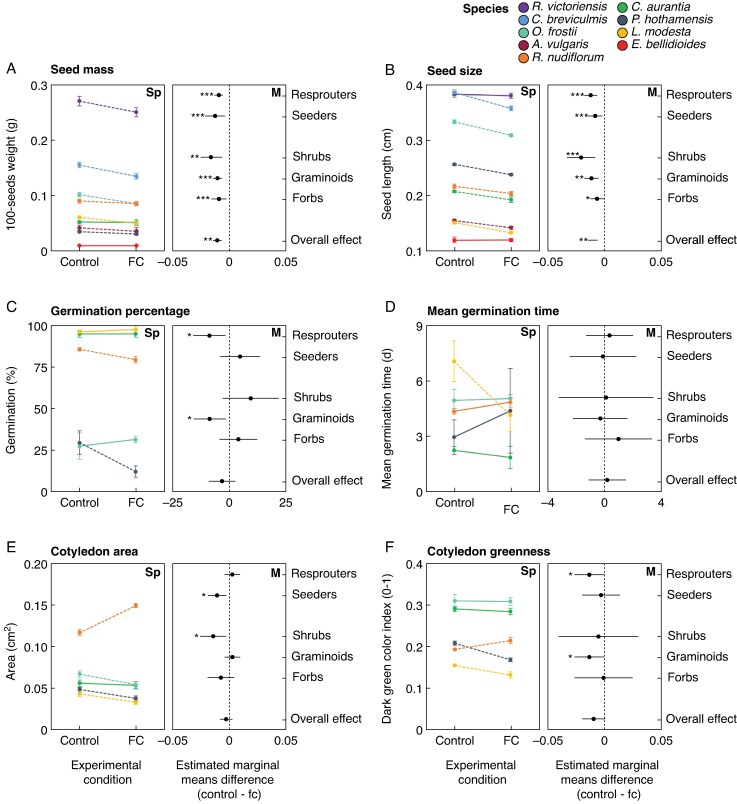
Effects of future climate treatment (FC) during the seed development stage on (A) seed mass, (B) seed size, (C) germination percentage, (D) germination time, (E) cotyledon area and (F) cotyledon greenness. Left panels (Sp) show species-specific responses where the line connecting the dots of the same colour shows the direction of change and the statistical significance, with the dashed line being *P* < 0.05. The right panels (M) show the differences between the estimated marginal means (control – treatment with 95 % CI) for the different life form and regeneration strategy categories (see Supplementary Data Tables S2 and S3).

Future climate during the seed development stage led to a decrease in seed mass and seed size (Supplementary Data Table S2). This decrease was observed in all categories of regeneration strategy and life form (Table S3 and Fig. 3A, B). However, there were some species-specific responses where *Erigeron bellidioides* and *Craspedia aurantia* showed no variation in seed mass, and *E. bellidioides* and *Ranunculus victoriensis* showed no variation in size between experimental conditions (Fig. 3A, B).

Overall, there was no effect of future climate treatment on the final proportion of germinated seeds and mean germination time (Supplementary Data Tables S2 and S3 and Fig. 3C, D). However, when considering the life form and recruitment strategy of the study species, the graminoids and resprouters showed a significant decrease in their germination percentage. These changes were driven by two members of the Poaceae family (*Rytosperma nudiflorum* and *Poa hothamensis*, Fig. 3C, D). No changes in mean germination time were observed when considering life form and regeneration strategy. However, one species significantly reduced its germination time (*Luzula modesta*, Fig. 3D).

Overall, there was no effect of future climate treatment on cotyledon greenness and cotyledon area (Supplementary Data Table S2 and S3 and Fig. 3E, F). However, when considering the life form and recruitment strategy of the study species, cotyledon area decreased in seeders and shrubs, and cotyledon greenness decreased in resprouters and graminoids (Fig. 3E, F). One species, *Rytosperma nudiflorum*, showed a contrasting response compared to the general patterns for both cotyledon area and greenness (Fig. 3E, F).

### Seed germination in the field

**Fig. 4.**
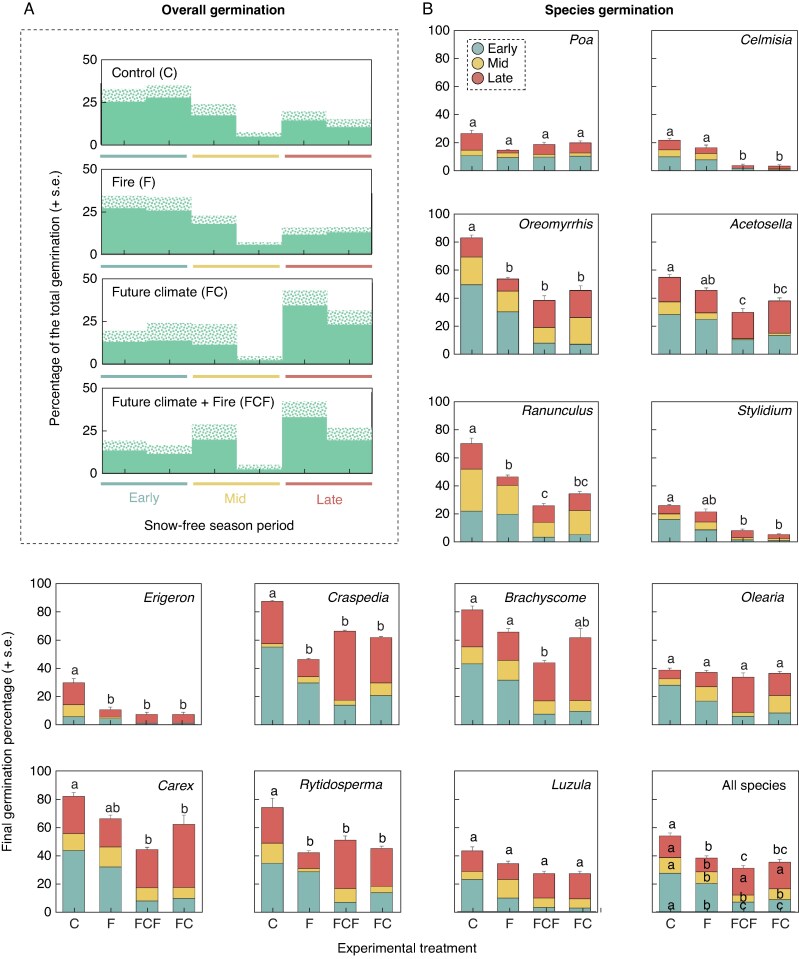
Germination patterns under the different experimental conditions (A). Values are averaged among sites and years. Species-specific germination response to treatments (B). For the individual species, letters above the error bars indicate significant differences in the final percentage of germination. For the all-species plot, letters within bars indicate differences for each period (see Supplementary Data Table S4 for results of *post hoc* tests).

Of the 12 740 seeds packed in mesh bags and sown in the field, 4105 germinated (32.55 %). Under control conditions, most germination occurred in the early period, but with continued germination events for the rest of the snow-free season. The experimental treatments affected the amount and timing of germination. In all the chambered plots (future climate and future climate and fire), germination decreased significantly, and the timing of germination shifted, with most seeds germinating later than in control conditions (Fig. 4A). The fire treatment also had a negative effect on germination, but to a lesser extent and did not cause changes in germination patterns (Fig. 4A and Table 2).

**Table 2.** Estimated intercepts (germination percentage) for linear models and their significance showing the effects of treatments on the germination of the different groups during the early, mid and late periods and on total germination. The values given for the treatments indicate their net effect on germination with respect to the control conditions (negative values indicate a decrease in germination and vice versa).

	Early			Mid			Late			Total germination		
	Estimate	s.e.	*P*-value	Estimate	s.e.	*P*-value	Estimate	s.e.	*P*-value	Estimate	s.e.	*P*-value
Overall												
Control	0.278	0.026	<0.001	0.103	0.017	<0.001	0.152	0.027	<0.001	0.533	0.056	<0.001
Future climate (FC)	−0.209	0.015	<0.001	−0.058	0.012	<0.001	0.024	0.019	0.5703	−0.243	0.024	<0.001
Fire (F)	−0.086	0.015	<0.001	−0.029	0.012	0.069	−0.058	0.019	0.009	−0.173	0.023	<0.001
FCF	−0.197	0.015	<0.001	−0.036	0.012	0.011	0.017	0.019	0.403	−0.216	0.023	<0.001
Resprouters												
Control	0.280	0.036	<0.001	0.079	0.022	0.002	0.168	0.038	0.005	0.527	0.080	0.008
Future climate (FC)	−0.193	0.022	<0.001	−0.043	0.016	0.211	0.030	0.025	0.641	−0.284	0.034	<0.001
Fire (F)	−0.079	0.022	0.010	−0.032	0.016	0.043	−0.073	0.026	0.026	−0.163	0.034	<0.001
FCF	−0.174	0.022	<0.001	−0.036	0.016	0.125	0.005	0.026	0.751	−0.228	0.034	<0.001
Seeders												
Control	0.274	0.039	<0.001	0.131	0.023	<0.001	0.133	0.041	0.005	0.539	0.086	0.009
Future climate (FC)	−0.226	0.022	0.002	−0.075	0.017	<0.001	0.017	0.027	0.921	−0.205	0.033	<0.001
Fire (F)	−0.094	0.022	<0.001	−0.025	0.017	0.426	−0.043	0.027	0.366	−0.183	0.033	<0.001
FCF	−0.221	0.022	<0.001	−0.036	0.017	0.135	0.030	0.027	0.668	−0.205	0.033	<0.001
Forbs												
Control	0.289	0.036	<0.001	0.117	0.023	0.001	0.161	0.038	0.008	0.568	0.078	<0.001
Future climate (FC)	−0.224	0.020	<0.001	−0.073	0.015	<0.001	0.000	0.023	0.958	−0.298	0.030	<0.001
Fire (F)	−0.098	0.020	<0.001	−0.036	0.015	0.071	−0.059	0.024	0.060	−0.193	0.029	<0.001
FCF	−0.214	0.020	<0.001	−0.050	0.015	0.004	0.012	0.024	0.958	−0.252	0.029	<0.001
Graminoids												
Control	0.253	0.050	0.002	0.092	0.032	0.01	0.157	0.053	0.010	0.502	0.110	0.008
Future climate (FC)	−0.175	0.028	0.002	−0.039	0.021	0.316	0.030	0.033	0.800	−0.184	0.042	0.001
Fire (F)	−0.056	0.028	<0.001	−0.036	0.021	0.247	−0.086	0.034	0.055	−0.178	0.042	0.002
FCF	−0.162	0.028	<0.001	−0.038	0.021	0.268	0.004	0.034	0.077	−0.196	0.042	<0.001
Shrubs												
Control	0.283	0.100	0.01	0.044	0.064	0.498	0.061	0.106	0.572	0.389	0.219	0.103
Future climate (FC)	−0.217	0.054	0.17	−0.017	0.041	0.876	0.183	0.065	0.024	−0.011	0.081	0.921
Fire (F)	−0.111	0.054	0.004	0.056	0.041	0.519	0.044	0.065	0.902	−0.050	0.081	0.976
FCF	−0.194	0.054	0.002	0.078	0.041	0.222	0.100	0.065	0.411	−0.017	0.081	0.963

Life form and regeneration strategy categories followed the overall trends described above (Table 2). However, species-specific responses were observed (Fig. 4B and Supplementary Data Table S4). Some species compensated for the reduction in germination in the early period by increasing their germination in the late period (e.g. *Brachyscome spathulata*, *Craspedia aurantia* and *Olearia Olearia frostii*). For other species, the reduction in germination in the early period was not compensated for later in the year (e.g. *L. modesta*, *Rytosperma nudiflorum*, *Ranunculus victoriensis*). Future climate treatment suppressed almost all germination during the mid period for a group of forbs (e.g. *Acetosella vulgaris*, *Stylidium armeria*, *Celmisia pugioniformis*). Furthermore, post-fire conditions reduced mid period germination in some species (e.g. *A. vulgaris*, *E. bellidioides*, *Rytosperma nudiflorum*). Finally, some species showed no response to the experimental treatments (e.g. *P. hiemata*).

### Seedling establishment

**Fig. 5.**
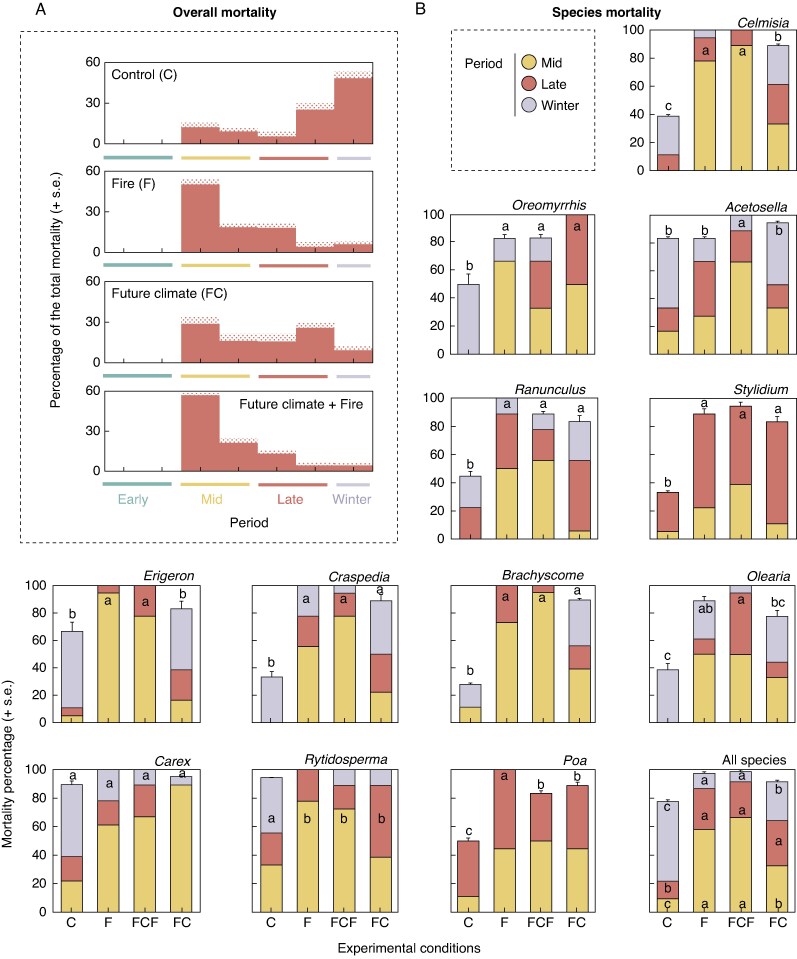
(A) Patterns of seedling mortality under different experimental treatments (values are averaged among species, sites and years) and (B) species-specific mortality percentages. For the individual species, letters within/above the error bars indicate differences between treatments at the end of the experiment. For the all-species plot, letters indicate differences for each period. For significance from *post hoc* tests, see Supplementary Data Table S7.

We recorded high rates of seedling mortality in both experimental years, with no differences between years (Table 3 and Supplementary Data Table S5). The experimental treatments affected the rates and timing of mortality events (Table 3 and Fig. 5). In general, mortality was higher under post-fire conditions (fire and future climate + fire plots), followed by future climate and control conditions. In the control conditions, most of the mortality occurred in the winter period (Table 3 and Fig. 5). In post-fire condition plots (i.e. fire and future climate + fire), most of the seedling mortality was recorded during the mid period for all species (>50 % of total mortality, Fig. 5A). In the chambered plots, mortality was more evenly distributed throughout the study period, but it was slightly higher in the mid period, too (Fig. 5A). When determining the mortality causes during the mid period, we found that smaller seedlings of graminoid species in post-fire conditions were more likely to die (Table S4).

**Table 3.** Probabilities of survival (estimates, s.e. and their significance) for the different life forms and regeneration strategies during the mid, late and post-winter periods. No mortality was recorded for the early period. Analysis was performed on the logit scale, but intervals in the table are back-transformed.

	Mid			Late			Post-winter		
	Prob.	s.e.	*P*-value	Prob.	s.e.	*P*-value	Prob.	s.e.	*P*-value
All species									
Control (C)	0.882	0.03	<0.001	0.716	0.04	<0.001	0.451	0.05	0.323
Fire (F)	0.333	0.05	0.001	0.118	0.03	<0.001	0.059	0.02	<0.001
Future climate (FC)	0.235	0.04	<0.001	0.069	0.03	<0.001	0.029	0.02	<0.001
FCF	0.608	0.05	0.031	0.255	0.04	<0.001	0.177	0.04	<0.001
Forbs									
Control (C)	0.924	0.03	<0.001	0.818	0.05	<0.001	0.546	0.06	0.461
Fire (F)	0.318	0.06	0.004	0.106	0.04	<0.001	0.061	0.03	<0.001
Future climate (FC)	0.197	0.05	<0.001	0.061	0.03	<0.001	0.046	0.03	<0.001
FCF	0.697	0.06	0.002	0.318	0.06	0.004	0.197	0.05	<0.001
Graminoids									
Control (C)	0.741	0.08	0.017	0.370	0.09	0.183	0.111	0.06	0.001
Fire (F)	0.370	0.09	0.183	0.074	0.05	0.001	0.000	0.00	0.988
Future climate (FC)	0.333	0.09	0.090	0.111	0.06	0.001	0.000	0.00	0.988
FCF	0.370	0.09	0.183	0.037	0.04	0.001	0.037	0.04	0.001
Shrub									
Control (C)	1.000	0.00	0.984	1.000	0.00	0.989	0.778	0.14	0.118
Fire (F)	0.333	0.16	0.327	0.333	0.16	0.327	0.222	0.14	0.118
Future climate (FC)	0.222	0.14	0.118	0.000	0.00	0.989	0.000	0.00	0.993
FCF	0.667	0.16	0.327	0.444	0.17	0.739	0.444	0.17	0.739
Seeders									
Control (C)	0.938	0.03	<0.001	0.854	0.05	<0.001	0.604	0.07	0.152
Fire (F)	0.292	0.07	0.005	0.104	0.04	<0.001	0.063	0.03	<0.001
Future climate (FC)	0.250	0.06	0.001	0.063	0.03	<0.001	0.063	0.03	<0.001
FCF	0.750	0.06	0.001	0.313	0.07	0.011	0.250	0.06	0.001
Resprouters									
Control (C)	0.833	0.05	<0.001	0.593	0.07	0.176	0.315	0.06	0.008
Fire (F)	0.370	0.07	0.060	0.130	0.05	<0.001	0.056	0.03	<0.001
Future climate (FC)	0.222	0.06	<0.001	0.074	0.04	<0.001	0.000	0.00	0.983
FCF	0.481	0.07	0.786	0.204	0.05	<0.001	0.111	0.04	<0.001

Regeneration strategy categories followed the overall mortality trend described above. However, life form-specific responses were observed, with shrubs having the lowest mortality rates in the experiment (Table 3). Species-specific responses were also observed (Supplementary Data Table S5 and Fig. 5B). Some species were more affected by future climate treatment than post-fire conditions (e.g. *Oreomyrrhis eripoda*). For some species, we did not record mortality during the winter period (e.g. *S. armeria* and *Poa hothamensis*). At the end of each experimental year, seedlings inside the chambers tended to be slightly shorter and had fewer leaves than those in the control plots, although these differences were not statistically significant (*P* = 0.06 and *P* = 0.07, respectively; Supplementary Data Table S6).

## DISCUSSION

Overall, we found negative and no effects of our experimental treatments on the life stages studied. In the following sections, we discuss the potential implications of a future warmer, drier and more fire-prone Australian alpine landscape for plant regeneration from seed.

### Experimental manipulations and microclimatic conditions

The modified OTCs created a warmer and drier microclimate in the organic and topsoil layers. The increase in soil surface temperature in chambered plots (+1.9 °C on average) is similar to the effect of hexagonal OTCs in the same study area (Camac *et al*., 2017) and is within the projected warming scenarios RCP 2.6 and 4.5 for high mountains (Hock *et al*., 2019). The decrease in soil VWC is similar to that reported for other traditional rain-out shelter experiments (Yahdjian and Sala, 2002, Kundel *et al*., 2018). Even during intense rainfall and seasonal peaks, soil moisture remained lower in chambered plots than in controls, probably due to reduced precipitation and enhanced evaporation.

Post-fire conditions did not affect soil VWC but increased soil surface and subsurface temperatures (Supplementary Data Table S1). This contrasts with the reported negative correlation between soil temperature and soil moisture, where an increase in temperature leads to a decrease in moisture due to increased evaporation (Legates *et al*., 2011). The absence of change in soil VWC could be explained by the lack of hydrological isolation of the plots and the size of the gaps being too small to induce high evaporative losses (i.e. the gap size was too small to create a gap effect in soil moisture). However, the recorded increase in soil temperature concurs with the reported effect of vegetation gaps on soil temperatures in other alpine environments (Aalto *et al*., 2013). Thus, while temperature effects appear realistic, our fire treatment may have underestimated moisture stress.

While our treatments were successful in creating warmer, drier and post-fire conditions, they may also have altered other unmeasured environmental variables, such as air humidity, CO_2_ concentrations or soil nutrients. Additionally, we did not assess potential changes in pollination dynamics or inbreeding under OTCs, which may also explain reductions in seed mass and viability. We recommend future studies account for such indirect effects of experimental use of OTCs and manipulative treatments.

Finally, the study period coincided with a multi-year La Niña event, with annual precipitation (1915 mm) well above the historical average (1359 mm for the 1990–2022 period, Falls Creek Meteorological Station, Bureau of Meteorology, www.bom.gov.au). Thus, germination and seedling establishment under control conditions may therefore reflect above average wet years.

### Seed development

The seed mass, size and germination rates recorded for seeds developed under control conditions are consistent with those previously reported for the study species (Sommerville *et al*., 2013, Morgan and Venn, 2017). To our knowledge, this is the first study to investigate the combined effects of higher temperatures and reduced water availability during seed development in alpine environments. Existing studies have only focused on the effects of either variable independently (Bernareggi *et al*., 2016, Gya *et al*., 2023, Notarnicola *et al*., 2023). Furthermore, no studies have focused on the seedling traits that we measured (i.e. cotyledon area and cotyledon greenness).

Higher temperatures and reduced water availability during seed development are known to have contrasting effects on seed mass and size. While increasing temperatures have generally resulted in an increase in seed mass and size (Wookey *et al*., 1995, Wagner and Reichegger, 1997, Totland and Alatalo, 2002, Wirth *et al*., 2010, Cui *et al*., 2017, Zhang *et al*., 2019), reductions in water availability have mostly resulted in a decrease in the size of these traits (Rosbakh *et al*., 2017, Peng *et al*., 2020). The reductions we observed suggest that, under climate change, warming effects may be constrained by water limitation (Mondoni *et al*., 2022).

Ecologically, seed mass is related to other traits, such as seed dispersal and seed persistence in soil seed banks (Saatkamp *et al*., 2019), with the detected changes having implications for alpine plant recruitment. The reduction in seed mass and size may increase dispersal distances, relevant in Australian alpine species with low dispersal (Morgan and Venn, 2017), but could also lead to deeper burial, potentially limiting germination in light-dependent seeds (Fernández-Pascual *et al*., 2021). A limitation of our study is that we did not record the timing of seed maturation, which may also be influenced by our treatments.

The overall neutral effect of a warmer and drier maternal environment on seed viability and germination speed is consistent with the existing literature (Mondoni *et al*., 2022). The general neutral effect we found on germination percentage concurs with the reported effect of higher temperatures during seed development for two Australian alpine forbs (Satyanti *et al*., 2021, Notarnicola *et al*., 2023) and several snowbed species in the European Alps (Bernareggi *et al*., 2016). However, as shown here, specific responses of species and functional groups are to be expected. The lack of effect of warm and dry maternal conditions on germination speed is consistent with that reported for several European snowbed species (Bernareggi *et al*., 2016) and the Australian alpine species *Wahlenbergia ceracea* (Notarnicola *et al*., 2023) and *Oreomyrrhys eripoda* (Satyanti *et al*., 2021). Interestingly, one species (*Luzula modesta*) significantly reduced its germination time, again suggesting species-specific responses. It is important to note that environmental conditions during seed development also affect the ability of seeds and emerged seedlings to cope with extreme climatic conditions via maternal effects (Donohue, 2009). We did not measure this adaptation, but we recognize that it must be considered when inferring the overall responses of alpine plants to climate change.

To our knowledge, this is the first study to measure cotyledon area and greenness in response to changing environmental conditions during seed development. The observed negative trends in both traits (with species- and group-specific responses) suggest negative consequences for seedling establishment. As key organs for early photosynthesis and energy supply, cotyledons play a crucial role in the establishment of seedlings (Fenner and Thompson, 2005, Hanley and May, 2006). Further studies are needed to better understand their role in alpine seedling establishment under climate change.

### Seed germination in the field

The overall germination rate recorded in the experiment (34.5 %) coincides with that reported in other seed-sowing field experiments in alpine environments (35 %: Margreiter *et al*., 2021; and 40 %: Mondoni *et al*., 2015). Furthermore, the germination pattern we found under control conditions (Fig. 4) is in line with the reported rapid onset of germination in spring after snowmelt in alpine environments but with a notable temporal spread of germination events throughout the rest of the snow-free season (Körner, 2021).

The reduction in the final percentage of germination under warmer and drier conditions contrasts with the reported effect of higher temperatures, which typically leads to an increase in germination (Fernández-Pascual *et al*., 2021). However, our results concur with the reported negative effect of reduced water availability on alpine seed germination (Walder and Erschbamer, 2015, Fernández-Pascual *et al*., 2021, Rosbakh *et al*., 2022, Gya *et al*., 2023). This suggests that changes in water availability will determine the net effect of warming conditions on alpine species germination. The detected shift in germination timing under warmer and drier conditions (from the early to the late period) may reflect inhibition of germination due to unfavourable environmental conditions; however, the induction of secondary dormancy is also possible, as primary dormancy had been broken by 8 weeks of cold stratification and the OTCs provided higher temperatures in autumn. A similar pattern in seedling emergence has been reported for the Australian Alps, where in a cool and wet year, most seedlings emerged in the spring, but in a dry and hot year, seedling emergence was partially suppressed in the spring and delayed until later in the snow-free season (Venn and Morgan, 2009).

The species-specific responses in germination to the fire treatment that we found are consistent with the reported changes in the diversity of emergent seedlings from soil seed banks following natural fires in Australian alpine landscapes (Wahren *et al*., 2001, Williams *et al*., 2008, 2012, Walsh and McDougall, 2015), and with the results of experimental studies investigating the effects of fire on alpine seed germination from the soil seed bank (Vázquez-Ramírez and Venn, 2023), where germination responses to fire were species-specific. Although it has been suggested that the seeds of seeder species are more tolerant to heat shock and have a greater heat-stimulated germination response than resprouters (Paula and Pausas, 2008), our classification of regeneration strategy did not explain which species were unaffected by the fire treatment (Fig. 4 and Supplementary Data Table S7).

From an ecological perspective, the reduced rates in germination as a consequence of warmer and drier conditions (expected to become the prevailing condition in Australian alpine environments) could increase the density of soil seed banks, where seeds will remain until favourable conditions for germination are met. Consequently, meaningful recruitment events in alpine landscapes will become more distinct and intermittent, occurring only in wet years. Over time, species with high moisture requirements for germination could be outcompeted by more robust alpine species, clonal taxa or lowland species that are less sensitive to soil moisture (Walder and Erschbamer, 2015, Rosbakh *et al*., 2017). If fire occurs before a wet year, species-specific fire responses could further alter plant community composition, favouring fire-tolerant species (e.g. shrubs, some graminoids) over fire-sensitive ones (e.g. most forbs). However, other factors such as intraspecific plasticity (e.g. seeds developed under drier conditions have a better germination capacity under water stress conditions; see Gya et al., 2023) may help some sensitive species persist. Further studies are needed to identify alpine species with high soil moisture requirements to guide conservation strategies.

The shift in germination time that we found under chambered plots coincides with the reported increase in autumn germination in the European Alps as a consequence of climate change (Mondoni *et al*., 2012, Orsenigo *et al*., 2015). However, it is necessary to mention that the drivers of these shifts are different. Here, the shift is a consequence of warmer spring–summer conditions, and in the above-mentioned studies, the increase in germination was driven by higher autumn temperatures after seed dispersal (promoting the germination of non-dormant seeds). Together, these results suggest that in the future, a greater number of seedlings will be exposed to harsh pre-winter alpine conditions, such as frost and soil freeze/thaw cycles, with unknown effects on seedling survival. In addition, existing studies have shown that the frost tolerance of alpine seedlings is not particularly strong compared to lowland species (Rosbakh *et al*., 2020).

In terms of our methods, the disturbance created during each monitoring event may have increased germination rates, as alpine germination can be promoted by soil disturbance (Hoyle *et al*., 2013) and light exposure (Fernández-Pascual *et al*., 2021). This should be considered when comparing our results with future studies. A limitation of our study is that we did not assess the condition of seeds collected at the end of the experiment, making it difficult to distinguish between seed mortality and dormancy. While mesh bags have been associated with fungal contamination that can increase seed mortality (Van Mourik *et al*., 2005), we did not observe any signs of fungal infection.

### Seedling establishment

The establishment rates we found under control conditions (45 %) are lower than those reported for glacier foreland plants in the Italian Alps (60–75 %; Mondoni et al., 2015), similar to those reported for an Australian forb (40–50 %; Geange *et al*., 2020) and natural seedling recruitment rates in the Australian Alps (30–56 %; Venn and Morgan, 2009) and higher than those reported in a seed-sowing experiment in the central European Alps (16 %; Margreiter *et al*., 2021).

The overall negative effect of the microclimate created by the chambers on seedling survival is consistent with the combined reported effects in the literature of reduced water availability and higher temperatures on seedling establishment (Vázquez-Ramírez and Venn, 2021). Similarly, other studies suggest that seedling mortality of alpine species due to drought stress is as important to survival rates as the mortality caused by cold conditions during winter (Forbis, 2003, Marcante *et al*., 2012). Furthermore, high mortality rates of *Aciphylla glacialis*, a species present in our study area, have been associated with higher temperatures and low soil moisture conditions (Geange *et al*., 2020). It has been suggested that the high soil moisture requirement for alpine seed germination is a strategy to reduce seedling mortality during hot and dry alpine summers (Körner, 2021).

The increase in seedling mortality we found in post-fire conditions may be due to the temperatures reached in bare soil patches during the hottest days of the year (up to 44.84 °C), which were similar to the reported heat tolerance of European alpine seedlings (around 46 °C; Marcante *et al*., 2012). Mortality may also reflect the lack of facilitative effects of adult plants, which are known to support seedling survival in alpine environments (Körner, 2021). While our results contrast with reports of positive recruitment in competition-free gaps (Margreiter *et al*., 2021), it is worth noting that the gap size in that study (9 cm) was smaller than ours (25 cm), suggesting that gap size may be a key factor influencing seedling establishment.

Ecologically, the different establishment rates that we found in the control and the future climate plots suggest that in a future warmer and drier alpine climate, seedling recruitment could be restricted only to years with suitable conditions (e.g. wet years such as those associated with La Niña weather patterns). However, if this type of weather event occurs after a year with a fire event, there could be a potential bottleneck in plant recruitment, as seedlings that emerge could quickly die from heat and drought stress in the bare soil. Should this occur, plant regeneration from seed may only occur after the resprouting species have created the necessary microclimatic conditions for seedling establishment, leading to possible changes in the diversity and composition of alpine plant communities. Additionally, the establishment rates that we found for the shrub *Olearia frostii* under post-fire conditions were higher compared to other life forms (although significantly lower than in control conditions, Supplementary Data Table S7). This is consistent with reported successful seedling establishment of other shrub species in post-fire conditions and higher temperatures (Camac *et al*., 2017), which may contribute to the predicted shift from alpine herbfields to shrublands in the Australian alpine landscape (Camac *et al*., 2017, Geange *et al*., 2020).

We monitored seedling survival and growth for 10 months (January to November), the period when most seedling mortality occurs in alpine species (Körner, 2021). However, longer-term studies are needed to assess the full impact of environmental changes in alpine plant populations. Further research should also investigate the heat and drought tolerance of Australian alpine seedlings, as our results suggest that these are key stressors for future establishment. Additionally, we planted the experimental seedlings in late spring to match reported seedling emergence patterns in our study region (Venn and Morgan, 2009), but our germination experiment revealed shifts in germination timing that need to be taken into account in future studies.

### Combined effects

Most studies on the response of alpine seeds and seedlings to climate change have focused on single environmental factors, particularly warming (Vázquez-Ramírez and Venn, 2021, Mondoni *et al*., 2022). In our study, the combined effects of warmer and drier conditions differed from those reported for temperature alone. When applied together, future climate and fire treatments produced different responses in germination and seedling survival across species and functional groups, highlighting the complexity of recruitment under multiple stressors. This suggests that the most impactful climate change effects arise from interacting environmental factors. We emphasize the need for future research to investigate these combined effects when assessing early life-history responses in alpine plants.

## CONCLUSION

We show that climate change will affect plant recruitment from seed in Australian alpine environments. Simulated changes in environmental factors affected the early life-history stages studied differently, but overall, negative and no effects were found. Warmer conditions and reduced water availability had a more pronounced negative effect on seed germination and seedling development. Post-fire conditions strongly affected seedling establishment and, to a lesser extent, germination. When the two experimental treatments were combined (future climate + fire), their effects on germination and seedling survival were not consistently more negative than either treatment alone, with responses varying across functional groups and species, suggesting complex effects of multiple stressors on recruitment. The potential implications of our results for plant regeneration in Australian alpine environments are: (1) a reduction in seed mass and size and cotyledon area (and possibly cotyledon greenness) as the climate becomes warmer and drier, with implications for germination and seedling establishment; (2) a reduction in germination in hot and dry years, which will be the new rule in the future, and more pronounced recruitment events in years with suitable conditions (e.g. wet and cool years such as those associated with La Niña weather patterns); (3) a shift of an important proportion of germination of drought-sensitive species from spring to autumn, exposing seedlings to harsh pre-winter environmental conditions; and (4) reduced seedling establishment in a future warmer and drier climate, with strong negative effects on recruitment into vegetation gaps created by fire or other factors. Finally, we emphasize that for a complete understanding of the effects of climate change on the early life-history stages of alpine plants, other factors that we did not consider in this study, such as intraspecific effects and phenotypic plasticity, need to be considered.

## Supplementary Material

mcaf132_Supplementary_Data

## SUPPLEMENTARY MATERIALS

Supplementary data are available at *Annals of Botany* online and consist of the following. Table S1: Microclimate conditions for experimental treatments. Table S2: ANOVA results for seed and seedling trait responses during seed development stage. Table S3: Trait differences between treatments with 95 % CIs during the seed development stage. Table S4: Pairwise germination differences (final and periodic) by treatment and species. Table S5: Pairwise comparisons of seedling survival probability (late period) across treatments and species. Table S6: Pairwise comparisons of seedling height and leaf number. Table S7: Pairwise comparisons of final germination (%) under post-fire conditions by functional group. Figure S1: Microclimate conditions for experimental treatments. Database S1: Raw data supporting the results of the experiment.
