# Supplementary material for: Climate change may alter seed and seedling traits and shift germination and mortality patterns in alpine environments
Source: Ann Bot. 2025 Jun 23;136(3):651–67. doi: 10.1093/aob/mcaf132 (PMC12455718; doi:10.1093/aob/mcaf132)
Supplement: mcaf132_Supplementary_Data [file mcaf132_supplementary_data.zip › Supplementary_materials.pdf]

Supplementary materials

Climate Change May Alter Seed and Seedling Traits and Shift Germination and Mortality Patterns in Alpine Environments

Jerónimo Vázquez-Ramírez[1*] and Susanna E. Venn[1]

[1] School of Life and Environmental Sciences, Deakin University, Australia 3125

[*] For correspondence: j.ramirez@research.deakin.edu.au

The following supplementary materials are available:

Tables

- Table S1. Microclimate conditions for experimental treatments.
- Table S2. ANOVA results for seed and seedling trait responses during seed development stage
- Table S3. Trait differences between treatments with 95% CIs during the seed development stage.
- Table S4. Pairwise germination differences (final and periodic) by treatment and species.
- Table S5. Pairwise comparisons of seedling survival probability (late period) across treatments and species.
- Table S6. Pairwise comparisons of seedling height and leaf number
- Table S7. Pairwise comparisons of final germination (%) under post-fire conditions by functional group.

Figures

- Figure S1. Microclimate conditions for experimental treatments.

Database

- Database S1. Raw data supporting the results of the experiment.

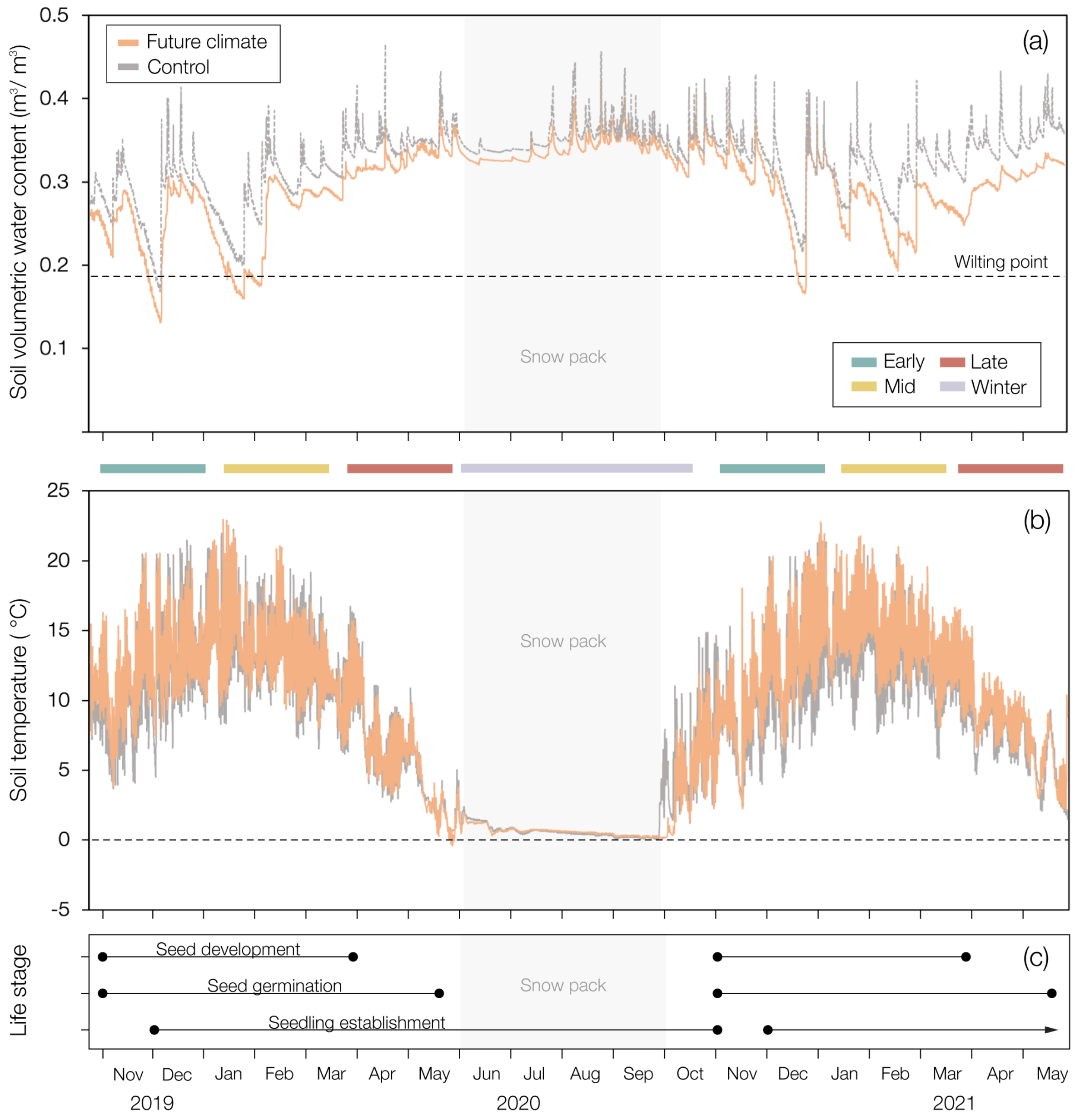

Figure S1. Mean soil volumetric water content (a) and soil temperature (b) at -3 cm in control (*n*=3, one per site) and chambered plots (*n*=3, one per site) during the study period. The bottom plot (c) shows the experimental timeline.

Table S1. Effects of the future climate (i.e., modified open-top chamber) and fire (i.e., bare soil gap) treatments on soil volumetric water content (10 cm below the soil surface), soil temperature (1-3 cm below the surface) and soil surface temperature. Values are averaged between sites. See the main manuscript for a detailed definition of study periods (early, mid and late).

	**Soil volumetric water content (m^3/m^3)**				**Soil temperature (°C)**				**Soil surface temperature (°C)**			
**Period**	**Early**	**Mid**	**Late**	**Overall**	**Early**	**Mid**	**Late**	**Overall**	**Early**	**Mid**	**Late**	**Overall**
2020-2021												
Control	0.2905	0.2685	0.3302	0.2964	14.01	13.40	8.01	11.81	15.50	15.01	7.47	12.66
Future climate	0.2577	0.2249	0.3001	0.2609	15.60	15.60	8.77	13.32	16.40	16.30	9.25	13.98
Fire	0.2908	0.2679	0.3303	0.2963	14.50	14.00	8.40	12.30	15.80	15.70	7.99	13.16
Control - Future climate	**-12.3%**	**-16.7%**	**-10%**	**-12%**	**+1.59**	**+2.20**	**+0.76**	**+1.52**	**+0.90**	**+1.29**	**+1.78**	**+1.32**
Control - Fire	**+0.2%**	**-0.3%**	**+0.01%**	**-0.7%**	**+0.49**	**+0.60**	**+0.39**	**+0.49**	**+0.30**	**+0.69**	**+0.52**	**+0.50**
2021-2022												
Control	0.3209	0.3091	0.3417	0.3239	11.90	13.01	8.70	11.20	12.58	13.02	8.60	11.40
Future climate	0.3002	0.2667	0.2922	0.2864	14.40	15.08	10.05	13.18	15.25	15.90	10.50	13.88
Fire	0.3206	0.3082	0.3409	0.3232	12.70	13.50	9.10	11.77	13.12	13.56	9.04	11.91
Control - Future climate	**-7%**	**-13.8%**	**-14.5%**	**-12.60%**	**+2.50**	**+2.07**	**+1.35**	**+1.97**	**+2.67**	**+2.88**	**+1.90**	**+2.48**
Control - Fire	**-0.1%**	**-0.3%**	**-0.4%**	**-0.3%**	**+0.80**	**+0.49**	**+0.40**	**+0.56**	**+0.54**	**+0.54**	**+0.44**	**+0.51**

Table S2. Anova-summary table (Type III) of the linear models testing the effects of the future climate treatment (modified open-top chambers) during seed development on seed mass (g), seed size (cm), final germination percentage (FGP, %), mean germination time (MGT, days), cotyledon area (cm^2) and cotyledon greenness (DGCI).

Treatment	Chi-square	df	*p* value
**Seed mass**			
Future Climate (FC)	28.091	1	**<0.001**
Life form x FC	0.577	2	0.749
Regeneration strategy x FC	1.216	1	0.270
**Seed size**			
Future Climate	24.911	1	**<0.0001**
Life form x FC	1.957	2	0.376
Regeneration strategy x FC	0.468	1	0.494
**Germination (FPG)**			
Future Climate	0.117	1	0.732
Life form x FC	7.173	2	**0.028**
Regeneration strategy x FC	5.892	1	**0.015**
**Germination (MGT)**			
Future Climate	1.301	1	0.254
Life form x FC	1.447	2	0.485
Regeneration strategy x FC	0.026	1	0.872
**Cotyledon area**			
Future Climate	0.849	1	0.357
Life form x FC	7.006	2	**0.030**
Regeneration strategy x FC	6.330	1	**0.012**
**Cotyledon greenness**			
Future Climate	3.441	1	0.064
Life form x FC	17.617	2	**<0.001**
Regeneration strategy x FC	23.235	1	**<0.001**

Table S3. Estimated marginal means (with 95% confidence intervals) comparing control and future climate treatments using linear models for seed mass (g), seed size (cm), mean germination time (days), germination percentage (%), cotyledon area (cm²), and cotyledon greenness (dark green colour index; DGCI). Negative values indicate a decrease in the response variable under future climate conditions.

	Estimate	SE	df	Lower CI	Upper CI	*t* ratio	*p* value	Estimate	SE	df	Lower CI	Upper CI	*t* ratio	*p* value
				**Seed mass**							**Seed size**			
**Life form**														
Forb	-0.009	0.003	69	-0.003	-0.016	-2.762	0.007	-0.007	0.003	438	0.000	-0.013	2.107	0.035
Graminoid	-0.010	0.003	69	-0.005	-0.016	-3.624	0.001	-0.012	0.003	438	-0.006	-0.018	3.729	<.0001
Shrub	-0.016	0.006	69	-0.005	-0.028	-2.881	0.005	-0.021	0.006	438	-0.009	-0.034	3.344	<.0001
**Regeneration strategy**														
Seeder	-0.013	0.003	70	-0.006	-0.019	-3.823	0.0003	-0.008	0.003	439	-0.002	-0.015	2.623	0.009
Sprouter	-0.009	0.003	70	-0.004	-0.014	-3.722	0.0004	-0.012	0.003	439	-0.007	-0.018	4.349	<.0001
				**Mean Germination Time**							**Germination Percentage**			
**Life form**														
Forb	0.963	1.182	39	3.35	-1.43	-0.815	0.420	3.93	4.14	39	12.29	-4.44	0.95	0.35
Graminoid	-0.321	0.965	39	1.63	-2.27	0.333	0.741	-8.81	3.54	32	-1.61	-16.01	-2.489	0.02
Shrub	0.073	1.672	39	3.46	-3.31	-0.044	0.965	9.64	6.13	32	22.1	-2.84	1.574	0.13
**Regeneration strategy**														
Seeder	-0.142	1.176	40	2.24	-2.52	-0.121	0.904	4.82	4.35	33	13.67	-4.03	1.109	0.28
Sprouter	0.331	0 0.832	40	2.01	-1.35	0.397	0.693	-8.81	3.55	33	-1.58	-16.03	-2.48	0.02
				**Cotyledon Area**							**Dark Colour Green Index**			
**Life form**														
Forb	-0.007	0.006	92	0.005	-0.019	1.198	0.234	-0.001	0.012	92	-0.022	0.025	0.110	0.913
Graminoid	0.003	0.003	92	0.010	-0.004	-0.875	0.384	-0.014	0.007	92	0.000	-0.027	2.034	0.045
Shrub	-0.014	0.006	92	-0.002	-0.026	2.378	0.020	-0.006	0.012	92	-0.017	0.030	0.514	0.609
**Regeneration strategy**														
Seeder	-0.011	0.004	93.000	-0.002	-0.019	2.533	0.013	-0.004	0.008	93.000	-0.013	-0.020	0.443	0.659
Sprouter	0.003	0.003	93.000	0.010	-0.004	-0.876	0.383	-0.014	0.007	93.000	0.000	0.027	2.045	0.044

Table S4. Within species estimated marginal means pairwise comparison for final and early, mid, late periods germination percentage. Values represent the difference between each comparison group and the reference group. FC = future climate treatment, FC_F= future climate + fire.

	**EARLY**			**MID**			**LATE**			**FINAL**		
**Contrasts**	**Estimate**	**SE**	***p* value**	**Estimate**	**SE**	***p* value**	**Estimate**	**SE**	***p* value**	**Estimate**	**SE**	***p* value**
Acetosella vulgaris												
Control-FC	0.178	0.05	0.002	0.081	0.04	0.170	-0.011	0.06	0.998	0.248	0.08	0.011
Control-FC_Fire	0.148	0.05	0.015	0.074	0.04	0.244	-0.056	0.06	0.815	0.167	0.08	0.161
Control-Fire	0.033	0.05	0.906	0.044	0.04	0.677	0.015	0.06	0.995	0.093	0.08	0.655
FC-FC_Fire	-0.030	0.05	0.932	-0.007	0.04	0.998	-0.044	0.06	0.896	-0.081	0.08	0.739
FC-Fire	-0.144	0.05	0.019	-0.037	0.04	0.787	0.026	0.06	0.977	-0.156	0.08	0.212
FC_Fire-Fire	-0.115	0.05	0.093	-0.030	0.04	0.878	0.070	0.06	0.681	-0.074	0.08	0.791
Brachyscome spathulata												
Control-FC	0.356	0.05	<.0001	0.026	0.04	0.924	0.005	0.07	1.000	0.388	0.09	<.0001
Control-FC_Fire	0.337	0.05	<.0001	0.041	0.04	0.745	-0.169	0.07	0.047	0.209	0.08	0.057
Control-Fire	0.115	0.05	0.111	-0.020	0.04	0.962	0.075	0.07	0.657	0.170	0.08	0.168
FC-FC_Fire	-0.019	0.05	0.982	0.015	0.04	0.984	-0.174	0.07	0.039	-0.178	0.08	0.136
FC-Fire	-0.241	0.05	<.0001	-0.046	0.04	0.671	0.070	0.07	0.702	-0.217	0.08	0.043
FC_Fire-Fire	-0.222	0.05	0.000	-0.061	0.04	0.414	0.244	0.06	0.001	-0.039	0.08	0.962
Carex breviculmis												
Control-FC	0.239	0.06	0.001	0.139	0.05	0.023	-0.133	0.08	0.313	0.244	0.10	0.063
Control-FC_Fire	0.272	0.06	0.000	0.056	0.05	0.663	-0.022	0.08	0.992	0.306	0.10	0.011
Control-Fire	0.000	0.06	1.000	0.128	0.05	0.044	0.061	0.08	0.859	0.189	0.10	0.219
FC-FC_Fire	0.033	0.06	0.946	-0.083	0.05	0.317	0.111	0.08	0.477	0.061	0.10	0.925
FC-Fire	-0.239	0.06	0.001	-0.011	0.05	0.996	0.194	0.08	0.059	-0.056	0.10	0.942
FC_Fire-Fire	-0.272	0.06	0.000	0.072	0.05	0.447	0.083	0.08	0.703	-0.117	0.10	0.634
Celmisia pugioniformis												

Control-FC	0.130	0.05	0.044	0.052	0.04	0.559	0.056	0.06	0.815	0.237	0.08	0.017
Control-FC_Fire	0.133	0.05	0.036	0.056	0.04	0.500	0.056	0.06	0.815	0.244	0.08	0.013
Control-Fire	0.100	0.05	0.179	0.030	0.04	0.878	0.011	0.06	0.998	0.141	0.08	0.295
FC-FC_Fire	0.004	0.05	1.000	0.004	0.04	1.000	0.000	0.06	1.000	0.007	0.08	1.000
FC-Fire	-0.030	0.05	0.932	-0.022	0.04	0.944	-0.044	0.06	0.896	-0.096	0.08	0.625
FC_Fire-Fire	-0.033	0.05	0.906	-0.026	0.04	0.914	-0.044	0.06	0.896	-0.104	0.08	0.566
Craspedia aurantia												
Control-FC	0.411	0.06	<.0001	-0.011	0.05	0.996	-0.189	0.08	0.071	0.211	0.10	0.138
Control-FC_Fire	0.344	0.06	<.0001	-0.067	0.05	0.518	-0.022	0.08	0.992	0.256	0.10	0.047
Control-Fire	0.244	0.06	0.000	-0.011	0.05	0.996	0.178	0.08	0.100	0.411	0.10	0.000
FC-FC_Fire	-0.067	0.06	0.687	-0.056	0.05	0.663	0.167	0.08	0.138	0.044	0.10	0.969
FC-Fire	-0.167	0.06	0.031	0.000	0.05	1.000	0.367	0.08	<.0001	0.200	0.10	0.175
FC_Fire-Fire	-0.100	0.06	0.348	0.056	0.05	0.663	0.200	0.08	0.049	0.156	0.10	0.387
Erigeron bellidioides												
Control-FC	0.022	0.05	0.969	0.059	0.04	0.442	0.063	0.06	0.751	0.144	0.08	0.273
Control-FC_Fire	0.033	0.05	0.906	0.059	0.04	0.442	0.063	0.06	0.751	0.156	0.08	0.212
Control-Fire	0.004	0.05	1.000	0.056	0.04	0.500	0.081	0.06	0.570	0.141	0.08	0.295
FC-FC_Fire	0.011	0.05	0.996	0.000	0.04	1.000	0.000	0.06	1.000	0.011	0.08	0.999
FC-Fire	-0.019	0.05	0.982	-0.004	0.04	1.000	0.019	0.06	0.991	-0.004	0.08	1.000
FC_Fire-Fire	-0.030	0.05	0.932	-0.004	0.04	1.000	0.019	0.06	0.991	-0.015	0.08	0.998
Luzula modesta												
Control-FC	0.196	0.05	0.001	-0.011	0.04	0.992	-0.026	0.06	0.977	0.159	0.08	0.194
Control-FC_Fire	0.200	0.05	0.000	-0.007	0.04	0.998	-0.033	0.06	0.952	0.159	0.08	0.194
Control-Fire	0.130	0.05	0.044	-0.074	0.04	0.244	0.033	0.06	0.952	0.089	0.08	0.683
FC-FC_Fire	0.004	0.05	1.000	0.004	0.04	1.000	-0.007	0.06	0.999	0.000	0.08	1.000
FC-Fire	-0.067	0.05	0.530	-0.063	0.04	0.387	0.059	0.06	0.784	-0.070	0.08	0.816
FC_Fire-Fire	-0.070	0.05	0.483	-0.067	0.04	0.335	0.067	0.06	0.717	-0.070	0.08	0.816
Olearia frostii												

Control-FC	0.217	0.05	0.000	0.017	0.04	0.975	-0.183	0.06	0.020	0.050	0.08	0.924
Control-FC_Fire	0.194	0.05	0.001	-0.078	0.04	0.205	-0.100	0.06	0.389	0.017	0.08	0.997
Control-Fire	0.111	0.05	0.111	-0.056	0.04	0.500	-0.044	0.06	0.896	0.011	0.08	0.999
FC-FC_Fire	-0.022	0.05	0.969	-0.094	0.04	0.082	0.083	0.06	0.551	-0.033	0.08	0.976
FC-Fire	-0.106	0.05	0.142	-0.072	0.04	0.265	0.139	0.06	0.125	-0.039	0.08	0.962
FC_Fire-Fire	-0.083	0.05	0.330	0.022	0.04	0.944	0.056	0.06	0.815	-0.006	0.08	1.000
Oreomyrrhis eriopoda												
Control-FC	0.415	0.05	<.0001	0.085	0.04	0.140	-0.056	0.06	0.815	0.444	0.08	<.0001
Control-FC_Fire	0.422	0.05	<.0001	0.007	0.04	0.998	-0.056	0.06	0.815	0.374	0.08	<.0001
Control-Fire	0.193	0.05	0.001	0.048	0.04	0.618	0.052	0.06	0.844	0.293	0.08	0.002
FC-FC_Fire	0.007	0.05	0.999	-0.078	0.04	0.205	0.000	0.06	1.000	-0.070	0.08	0.816
FC-Fire	-0.222	0.05	0.000	-0.037	0.04	0.787	0.107	0.06	0.325	-0.152	0.08	0.231
FC_Fire-Fire	-0.230	0.05	<.0001	0.041	0.04	0.734	0.107	0.06	0.325	-0.081	0.08	0.739
Poa hiemata												
Control-FC	0.011	0.05	0.996	0.019	0.04	0.966	0.048	0.06	0.871	0.078	0.08	0.766
Control-FC_Fire	0.004	0.05	1.000	0.015	0.04	0.982	0.048	0.06	0.871	0.067	0.08	0.839
Control-Fire	0.015	0.05	0.991	0.004	0.04	1.000	0.100	0.06	0.389	0.119	0.08	0.450
FC-FC_Fire	-0.007	0.05	0.999	-0.004	0.04	1.000	0.000	0.06	1.000	-0.011	0.08	0.999
FC-Fire	0.004	0.05	1.000	-0.015	0.04	0.982	0.052	0.06	0.844	0.041	0.08	0.957
FC_Fire-Fire	0.011	0.05	0.996	-0.011	0.04	0.992	0.052	0.06	0.844	0.052	0.08	0.916
Ranunculus victoriensis												
Control-FC	0.233	0.05	<.0001	0.196	0.04	<.0001	0.063	0.06	0.751	0.493	0.08	<.0001
Control-FC_Fire	0.215	0.05	0.000	0.130	0.04	0.006	0.063	0.06	0.751	0.407	0.08	<.0001
Control-Fire	0.070	0.05	0.483	0.096	0.04	0.074	0.122	0.06	0.215	0.289	0.08	0.002
FC-FC_Fire	-0.019	0.05	0.982	-0.067	0.04	0.335	0.000	0.06	1.000	-0.085	0.08	0.712
FC-Fire	-0.163	0.05	0.006	-0.100	0.04	0.058	0.059	0.06	0.784	-0.204	0.08	0.055
FC_Fire-Fire	-0.144	0.05	0.019	-0.033	0.04	0.835	0.059	0.06	0.784	-0.119	0.08	0.450
Rytidosperma nudiflorum												

Control-FC	0.274	0.05	<.0001	0.044	0.04	0.677	-0.044	0.06	0.896	0.274	0.08	0.004
Control-FC_Fire	0.207	0.05	0.000	0.096	0.04	0.074	-0.015	0.06	0.995	0.289	0.08	0.002
Control-Fire	0.059	0.05	0.626	0.119	0.04	0.016	0.141	0.06	0.117	0.319	0.08	0.001
FC-FC_Fire	-0.067	0.05	0.530	0.052	0.04	0.559	0.030	0.06	0.966	0.015	0.08	0.998
FC-Fire	-0.215	0.05	0.000	0.074	0.04	0.244	0.185	0.06	0.019	0.044	0.08	0.945
FC_Fire-Fire	-0.148	0.05	0.015	0.022	0.04	0.944	0.156	0.06	0.067	0.030	0.08	0.983
Stylidium armeria												
Control-FC	0.126	0.05	0.053	0.063	0.04	0.387	0.007	0.06	0.999	0.196	0.08	0.069
Control-FC_Fire	0.130	0.05	0.044	0.056	0.04	0.500	0.020	0.06	0.988	0.206	0.08	0.052
Control-Fire	0.080	0.05	0.371	0.026	0.04	0.914	-0.028	0.06	0.972	0.078	0.08	0.766
FC-FC_Fire	0.004	0.05	1.000	-0.007	0.04	0.998	0.013	0.06	0.997	0.009	0.08	0.999
FC-Fire	-0.046	0.05	0.784	-0.037	0.04	0.787	-0.035	0.06	0.945	-0.119	0.08	0.450
FC_Fire-Fire	-0.050	0.05	0.741	-0.030	0.04	0.878	-0.048	0.06	0.871	-0.128	0.08	0.382

Table S5. Within species pairwise comparison of estimated marginal means for the probability of seedling survival at the end of the snow-free season (late-period) in the different experimental conditions. Values are averaged across sites and plots.

Contrasts	Prob.	SE	*p* value
Acetosella vulgaris			
Control - Fire	0	0.149	1
Control - Future climate + Fire	0.333	0.149	0.1524
Control - Future climate	0.222	0.149	0.8126
Fire - Future climate + Fire	0.333	0.149	0.1524
Fire - Future climate	0.222	0.149	0.8126
Future climate + Fire - Future climate	-0.111	0.149	1
Brachyscome spathulata			
Control - Fire	0.333	0.149	0.1524
Control - Future climate + Fire	0.333	0.149	0.1524
Control - Future climate	0.111	0.149	1
Fire - Future climate + Fire	0	0.149	1
Fire - Future climate	-0.222	0.149	0.8126
Future climate + Fire - Future climate	-0.222	0.149	0.8126
Carex breviculmis			
Control - Fire	0.222	0.149	0.8126
Control - Future climate + Fire	0.222	0.149	0.8126
Control - Future climate	0.111	0.149	1
Fire - Future climate + Fire	0	0.149	1
Fire - Future climate	-0.111	0.149	1
Future climate + Fire - Future climate	-0.111	0.149	1
Celmisia pugioniformis			
Control - Fire	0.556	0.149	0.0013
Control - Future climate + Fire	0.556	0.149	0.0013
Control - Future climate	0.333	0.149	0.1524
Fire - Future climate + Fire	0	0.149	1
Fire - Future climate	-0.222	0.149	0.8126
Future climate + Fire - Future climate	-0.222	0.149	0.8126
Craspedia aurantia			
Control - Fire	0.667	0.149	0.0001
Control - Future climate + Fire	0.667	0.149	0.0001
Control - Future climate	0.444	0.149	0.0177
Fire - Future climate + Fire	0	0.149	1
Fire - Future climate	-0.222	0.149	0.8126
Future climate + Fire - Future climate	-0.222	0.149	0.8126
Erigeron bellidiodes			

Control - Fire	0.667	0.149	0.0001
Control - Future climate + Fire	0.667	0.149	0.0001
Control - Future climate	0.333	0.149	0.1524
Fire - Future climate + Fire	0	0.149	1
Fire - Future climate	-0.333	0.149	0.1524
Future climate + Fire - Future climate	-0.333	0.149	0.1524
Olearia frostii			
Control - Fire	0.556	0.149	0.0013
Control - Future climate + Fire	0.778	0.149	<.0001
Control - Future climate	0.333	0.149	0.1524
Fire - Future climate + Fire	0.222	0.149	0.8126
Fire - Future climate	-0.222	0.149	0.8126
Future climate + Fire - Future climate	-0.444	0.149	0.0177
Oreomyrrhis eriopoda			
Control - Fire	0.667	0.257	0.0596
Control - Future climate + Fire	0.667	0.257	0.0596
Control - Future climate	1	0.257	0.0007
Fire - Future climate + Fire	0	0.257	1
Fire - Future climate	0.333	0.257	1
Future climate + Fire - Future climate	0.333	0.257	1
Poa hothamensis			
Control - Fire	0	0.149	1
Control - Future climate + Fire	0	0.149	1
Control - Future climate	0	0.149	1
Fire - Future climate + Fire	0	0.149	1
Fire - Future climate	0	0.149	1
Future climate + Fire - Future climate	0	0.149	1
Ranunculus victoriensis			
Control - Fire	0.889	0.149	<.0001
Control - Future climate + Fire	0.667	0.149	0.0001
Control - Future climate	0.556	0.149	0.0013
Fire - Future climate + Fire	-0.222	0.149	0.8126
Fire - Future climate	-0.333	0.149	0.1524
Future climate + Fire - Future climate	-0.111	0.149	1
Rytidosperma nudiflorum			
Control - Fire	0.111	0.149	1
Control - Future climate + Fire	0.111	0.149	1
Control - Future climate	0.111	0.149	1
Fire - Future climate + Fire	0	0.149	1
Fire - Future climate	0	0.149	1
Future climate + Fire - Future climate	0	0.149	1
Stylidium armeria			
Control - Fire	0.222	0.149	0.8126

Control - Future climate + Fire	0.222	0.149	0.8126
Control - Future climate	0.222	0.149	0.8126
Fire - Future climate + Fire	0	0.149	1
Fire - Future climate	0	0.149	1
Future climate + Fire - Future climate	0	0.149	1

Table S6. Estimated marginal means and pairwise comparisons calculated from the linear models testing the effects of experimental conditions on seedlings number of leaves and height (cm) before winter snow cover. Values are averaged across species, sites and years.

**Leaf number**			
**Treatment**	**Estimate**	**SE**	***p* value**
Control	5.930	0.75	<.0001
Fire	6.450	1.07	<.0001
Future	5.430	1.07	<.0001
Future	4.500	0.69	<.0001
Pairwise – Leaf number			
Control - Fire	0.919	0.12	0.906
Control - Future climate + Fire	1.093	0.19	0.954
Control - Future climate	1.317	0.14	**0.056**
Fire - Future climate + Fire	1.189	0.23	0.806
Fire - Future climate	1.433	0.21	**0.067**
Future climate + Fire - Future climate	1.205	0.23	0.758
**Height (cm)**			
**Treatment**	**Est.**	**SE**	***p*-value**
Control	3.840	0.33	<.0001
Fire	3.160	0.51	<.0001
Future	2.460	0.50	<.0001
Future	3.430	0.40	<.0001
Pairwise – Height			
Control - Fire	0.830	0.57	0.47
Control - Future climate + Fire	1.665	0.67	**0.07**
Control - Future climate	0.457	0.40	0.66
Fire - Future climate + Fire	0.835	0.84	0.75
Fire - Future climate	-0.373	0.63	0.93
Future climate + Fire - Future climate	-1.208	0.74	0.37

Table S7. Within functional groups pairwise comparison of estimated marginal means for the final percentage of germination in post-fire experimental conditions. Values are averaged across sites and plots.

Post-fire conditions and germination						
	**Est.**	**SE**	**df**	**Lower CI**	**Upper CI**	***p* value**
**Regeneration strategy**						
Seeders	0.376	0.0856	0.123	0.19	0.562	0.001
Resprouters	0.344	0.0796	0.126	0.172	0.517	0.001
Contrast	0.0317	0.117	0.124	-0.222	0.285	0.791
**Life form**						
Forb	0.375	0.078	0.112	0.205	0.545	0.0005
Graminoid	0.324	0.110	0.112	0.083	0.565	0.013
Shrub	0.378	0.219	0.111	-0.104	0.860	0.1124
F-G	0.051	0.135	0.112	-0.311	0.413	0.9242
F-S	-0.003	0.232	0.111	-0.630	0.624	0.9999
G-S	-0.054	0.245	0.111	-0.715	0.607	0.9738
